# Amyloid precursor protein and its interacting proteins in neurodevelopment

**DOI:** 10.1042/BST20221527

**Published:** 2023-06-30

**Authors:** Dennis Dik-Long Chau, Laura Lok-Haang Ng, Yuqi Zhai, Kwok-Fai Lau

**Affiliations:** School of Life Sciences, Faculty of Science, The Chinese University of Hong Kong, Hong Kong SAR, China

**Keywords:** amyloid precursor protein, neurodevelopment, protein–protein interaction

## Abstract

Amyloid precursor protein (APP) is a key molecule in the pathogenesis of Alzheimer's disease (AD) as the pathogenic amyloid-β peptide is derived from it. Two closely related APP family proteins (APPs) have also been identified in mammals. Current knowledge, including genetic analyses of gain- and loss-of-function mutants, highlights the importance of APPs in various physiological functions. Notably, APPs consist of multiple extracellular and intracellular protein-binding regions/domains. Protein–protein interactions are crucial for many cellular processes. In past decades, many APPs interactors have been identified which assist the revelation of the putative roles of APPs. Importantly, some of these interactors have been shown to influence several APPs-mediated neuronal processes which are found defective in AD and other neurodegenerative disorders. Studying APPs–interactor complexes would not only advance our understanding of the physiological roles of APPs but also provide further insights into the association of these processes to neurodegeneration, which may lead to the development of novel therapies. In this mini-review, we summarize the roles of APPs–interactor complexes in neurodevelopmental processes including neurogenesis, neurite outgrowth, axonal guidance and synaptogenesis.

## Introduction to amyloid precursor protein

Amyloid precursor protein (APP) is one of the major interests in current research on Alzheimer's disease (AD) since the aberrant processing of APP contributes to the generation and accumulation of neurotoxic peptide amyloid-β (Aβ). Apart from its significant relevance in AD pathogenesis, APP also participates in various cellular activities which facilitate brain development and functions. A comprehensive understanding of the structure, physiological functions of APP and the interaction between APP and its interacting partners is crucial for the development of novel preventive and therapeutic strategies for AD.

**Figure 1.**
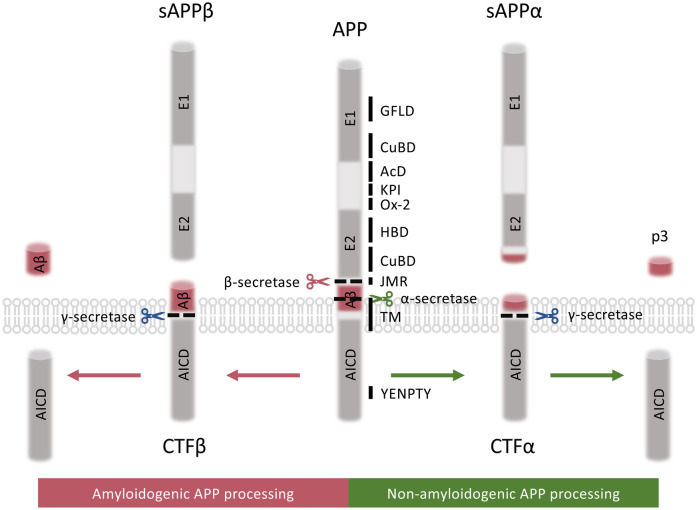
Schematic diagram to show APP structure and processing. The structure of APP and its non-amyloidogenic and amyloidogenic processing pathways are illustrated. The N-terminal ectoplasmic domain of APP consisted of E1 and E2 domains, with AcD connecting E1 and E2 and JMR connecting the ectodomain to TM. E1 domain constitutes a GFLD and a CuBD. E2 domain possesses a HBD and a CuBD. Ox-2 and KPI domain are also contained in the APP ectodomain. During non-amyloidogenic processing, APP is first cleaved by α-secretase to release sAPPα and membrane-bound CTFα. CTFα is then further cleaved by γ-secretase to produce the p3 fragment and AICD. During amyloidogenic pathway, APP is cleaved by β-secretase to generate sAPPβ and membrane-bound CTFβ, which is further cleaved by γ-secretase to generate Aβ and AICD. AcD, acidic domain; AICD, amyloid precursor protein intracellular domain; APP, amyloid precursor protein; Aβ, amyloid-β; CTFα, C-terminal fragment α; CTFβ, C-terminal fragment β; CuBD, copper-binding domain; E1, E1 domain; E2, E2 domain; GFLD, growth-factor-like domain; HBD, heparin-binding domain; JMR, juxtamembrane region; KPI, kunitz protease inhibitor domain; Ox-2, Ox-2 antigen domain; p3, p3 fragment; sAPPα, secreted APPα; sAPPβ, secreted APPβ; TM, transmembrane helix; YENPTY, tyrosine–glutamate–asparagine–proline–threonine–tyrosine domain.

APP is a type I transmembrane protein composed of a large N-terminal luminal region, a single-spanning transmembrane domain and a short C-terminal cytosolic tail [1,2]. Alternative splicing of the *APP* gene generates eight isoforms, with three major isoforms including APP_695_, APP_751_ and APP_770_ [3]. In the central nervous system, neurons predominantly express the APP_695_ isoform [4]. Two closely related APP-like proteins have been identified in mammals, namely APP-like protein 1 and 2 (APLP1 and APLP2). The N-terminal ectoplasmic domain of APP family proteins (APPs) mainly consisted of E1 and E2 domains, with the acidic domain (AcD) connecting E1 and E2 and the juxtamembrane region (JMR) connecting the ectodomain to the transmembrane helix (TM). In detail, the E1 domain constitutes a growth-factor-like domain (GFLD) and a copper-binding domain (CuBD). E2 domain possesses a heparin-binding domain (HBD) and a CuBD. The Ox-2 antigen domain (Ox-2) and the kunitz protease inhibitor (KPI) domain are also contained in the ectodomain of longer APP isoforms. (Ox-2 in APP_770_; KPI in APP_751_ and APP_770_) (Figure 1). The structure of APP resembles cell surface receptors with some putative extracellular ligands [4]. In addition, APP is able to homodimerize or heterodimerize with APLPs and other integral membrane proteins [4]. APP dimerization is dependent on the binding of copper to the HBD and CuBD in APPs [5–7]. On the cytosolic side, the APP intracellular domain (AICD) contains a conserved tyrosine–glutamate–asparagine–proline–threonine–tyrosine (YENPTY) motif that is crucial for APP intracellular sorting. Most AICD-interacting proteins contain a phosphotyrosine-binding (PTB) domain, including those in X11/APBA family and FE65/APBB family proteins that recognizes YENPTY motifs [8–10]. Some AICD-interacting proteins have been shown to influence APP processing [11–17].

APP is subjected to regulated intramembrane proteolysis (RIP) which yields secreted extracellular peptides and cytosolic fragments [1]. APP may be metabolized by two mutually exclusive pathways, namely amyloidogenic and non-amyloidogenic pathways (Figure 1). Non-amyloidogenic APP processing by α-secretase and γ-secretase releases secreted APPα (sAPPα) and membrane-bound C-terminal fragment α, which is further cleaved to generate the p3 fragment and AICD [19]. During amyloidogenic processing, stepwise cleavage of APP by β-secretase and γ-secretase produces the neurotoxic Aβ [19]. Under physiological conditions, the majority of APP is cleaved within the Aβ sequence through the non-amyloidogenic pathway, thus precluding the generation of Aβ. However, a shift towards the amyloidogenic pathway would contribute to an increase in Aβ generation and the pathogenesis in AD. Extracellular deposition of Aβ plaques on neurons is the key pathology hallmark of AD. Both APLP1 and APLP2 undergo RIP via a process similar to that in APP, releasing non-pathological p3-like fragments from APLP1 and p3- and Aβ-like fragments from APLP2 [18].

**Figure 2.**
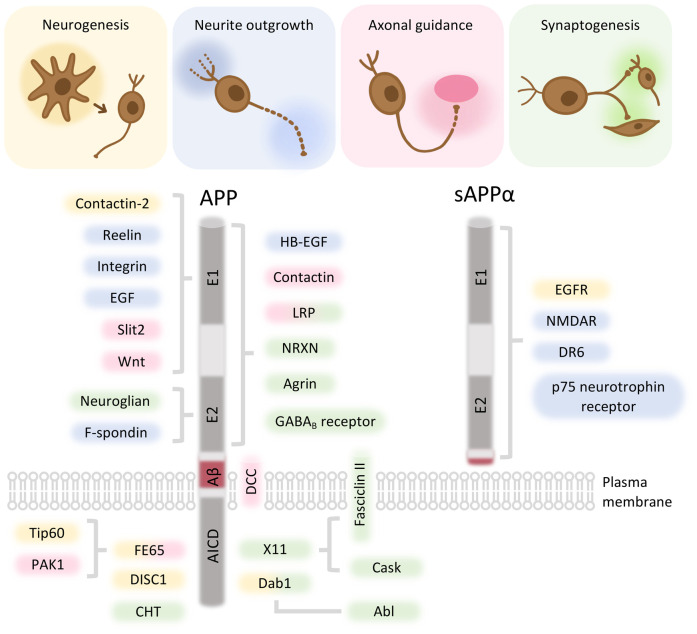
Summary of APP and its interactors in neurodevelopment. The domains or regions of APP for the binding to its interactors are indicated. The interactors that are associated with APP-mediated neurogenesis (yellow), neurite outgrowth (blue), axonal guidance (pink) and synaptogenesis (green) are shown. APP, amyloid precursor protein; sAPPα, secreted APPα; Aβ, amyloid-β; p3, p3 fragment; E1, E1 domain; E2, E2 domain; AICD, APP intracellular domain; EGF, epidermal growth factor; PAK1, p21-activated kinase 1; DISC1, disrupted-in-schizophrenia 1; HB-EGF, heparin-binding EGF-like growth factor; LRP, low-density lipoprotein receptor-related protein; NRXN, Neurexin; Dab1, Disabled-1; Cask, calcium/calmodulin-dependent serine protein kinase; EGFR, epidermal growth factor receptor; NMDAR, *N*-methyl-d-aspartic acid receptor; DR6, death receptor 6; Abl, Abl tyrosine kinase.

Despite its pathological role in neurodegeneration, mounting evidence suggests that APPs are crucial for neurodevelopment. APP is first expressed in the developing mouse cortex on embryonic day 9.5, when neurons start to differentiate [19], and its level increases continuously during development [20,21]. The physiological functions of the APPs have been investigated *in vivo* using single-, double- and triple-knockout mouse mutants. Notably, the knockout of only one of the APPs in mice results in subtle phenotypes such as reduced brain weight [22] and muscle weakness [23]. Meanwhile, *App*^−/−^/*Aplp2*^−/−^, *Aplp1*^−/−^/*Aplp2*^−/−^ double knockout and *App*^−/−^/*Aplp1*^−/−^/*Aplp2*^−/−^ triple-knockout mouse mutants show early postnatal lethality [24,25]. The triple-knockout mice display major morphological abnormalities with cortical dysplasias, a phenotype that resembles human type II lissencephaly [24]. These studies suggest the functional redundancy of APPs in brain development and postnatal survival [24]. As APPs consist of multiple protein-binding regions and domains, their functions in neurodevelopment are likely contributed, at least in part, by binding to their interactors. Understanding the physical and functional interactions between APP and its interactors is essential for defining their relationship in various signaling pathways and for predicting druggable targets. In this article, we summarize the roles of the interactors that interplay with APP during several major neurodevelopmental events (Figure 2).

## The interplay of APP and its interactors during neurodevelopment

### Neurogenesis

Neurogenesis is the formation of differentiated neurons from neural precursor cells (NPCs) and the integration of newly formed neurons into the pre-existing neural circuitry. Several findings imply that APP regulates neurogenesis in both developing and adult brains. Ma et al. [26] showed that contactin-2, also known as TAG-1, is a functional APP ligand that suppresses neurogenesis in the developing mouse brain. The effect of APP on neurogenesis has been shown to be associated with the cytosolic adaptor FE65. Abnormalities due to contactin-2 knockout can be reversed by the overexpression of AICD but not an AICD with a mutated FE65 binding site. The binding of contactin-2 to APP stimulates the generation of AICD, which has been shown to complex with FE65 and Tat interactive protein 60 kDa (Tip60) histone acetyltransferase and to modulate gene transactivation [27]. Some AICD-FE65-Tip60-regulated genes including stathmin [28], are implicated in various neuronal processes in adult neurogenesis [29]. Moreover, the AICD–FE65–Tip60 complex participates in neurogenesis through neuronal fate specification and activity-dependent neuroadaptation as Tip60 has been reported to be critical for these two processes [30,31].

The AICD–FE65 complex has also been found to negatively regulate adult neurogenesis, a process associated with hippocampal-mediated memory function [32]. Ghosal et al. [33] showed that adult neurogenesis is impaired in transgenic mice co-expressing AICD and FE65 [33]. Moreover, a synthetic peptide P33 disrupts the APP–FE65 interaction and enhances the memory and learning functions of APPswe/PS1dE9 mice, a common mouse model for studying familial AD, by ameliorating neuroinflammation [34]. Conversely, there are reports suggesting the importance of the APP–FE65 interaction in enhancing or preserving the memory function. The introduction of point mutation threonine 668 (T668) to alanine in APP, which precludes phosphorylation, is shown to prevent memory and synaptic impairments in a familial Danish dementia mouse model [35]. Of note, the phosphorylation of APP residue T668 abolishes the interaction between APP and FE65 [36,37]. Although this discrepancy needs to be resolved, currently available data support the role of the APP–FE65 interaction in adult neurogenesis.

Disabled-1 (Dab1) and disrupted-in-schizophrenia 1 (DISC1) are also AICD interactors implicated in neurogenesis. The stimulatory effect of APP on the migration of cortical neurons is eliminated by a Dab1 binding defective APP mutant. Moreover, overexpression of Dab1 rescues the migration defects caused by APP knockdown in the cortices of the embryos [38]. Likewise, APP knockdown disrupts the localization of DISC1 to the centrosome which results in the faulty migration of embryonic cortical neurons into the cortical plate [39]. Although it remains unknown how the interactions between APP and Dab1/DISC1 are co-ordinated, the abovementioned findings highlight the significance of APP in cortical migration during development [40].

Intriguingly, overmigration of cortical plate neurons is observed in *App*^−/−^/*Aplp1*^−/−^/*Aplp2*^−/−^ triple-knockout mice. The contradictory observation may be due to the difference between acute and chronic genetic depletion models used in different studies. In fact, Young-Pearse et al. [38] observed an increased APLP2 expression in APP knockout but not in APP knockdown animals. Besides, the APPs share several interactors with other type I transmembrane proteins at the Asn-Pro-X-Tyr (NPXY) motif including the members of the low-density lipoprotein receptors (LDLRs) and the Notch receptor family. For instance, APP interacts with reelin, a large secreted extracellular matrix glycoprotein that participates in many neuronal events including neuronal migration [41]. Mutation in the *reelin* gene results in motor dysfunction observed in *reeler* mouse [42]. Malformations of the cortical development have also been observed in the *reeler* mice, which exhibit inversion of the cortical plate [43]. Of note, *App*^−/−^/*Aplp1*^−/−^/*Aplp2*^−/−^ triple-knockout also results in abnormal neuronal positioning. Disorganized and reduced number of Cajal–Retzius (CR) cells is observed in *App*^−/−^/*Aplp1*^−/−^/*Aplp2*^−/−^ triple-knockout mice [24]. CR cells are reelin-secreting cells that modulate the architectural development of the cortical plate [44,45]. However, *App*^−/−^/*Aplp1*^−/−^/*Aplp2*^−/−^ triple-knockout does not impact the normal inside-out orientation of the cortical layers [24]. Additionally, APP-FE65 interaction is suggested to influence APPs effect on neuronal migration. FE65^−/−^/FE65-like 1 (FE65L1)^−/−^ double knockout mice display focal neuronal ectopia and loss of CR neurons, of which the phenotypes overlap with those observed in *App*^−/−^/*Aplp1*^−/−^/*Aplp2*^−/−^ triple-knockout mice [46].

The neurotrophic property of sAPPα has been long observed in multiple cell types. For instance, the addition of sAPPα promotes the proliferation of NPCs while the removal of sAPPα decreases the process. Intraventricular injection of recombinant sAPPα rescues the decline of neurogenesis in the subgranular layer of the dentate gyrus and subventricular zone in ageing mice [47]. As sAPPα co-localizes with NPCs expressing the epidermal growth factor receptor (EGFR), it has been suggested to function as a receptor for sAPPα [48]. Other putative sAPPα receptors have also been proposed. For example, sAPPα interacts with gamma-aminobutyric acid type B receptor (GBR) subunit 1a to modulate neurotransmission [49]. Evidence has shown that GBR signaling mediates hippocampal neurogenesis [50,51]. Moreover, it is suggested that the cell surface APP acts as a sAPPα receptor to the regulate survival of neuroblastoma cells [52]. These findings suggest that sAPPα participates in neurogenesis via multiple signaling pathways.

### Neurite outgrowth

Neurite outgrowth is an essential step in nervous system development, as it produces new projections for the wiring of neurons. APP has been shown to regulate neurite outgrowth. Both sAPPα and membrane-associated APP increase the average neurite length [2,53], while APP knockdown inhibits neurite outgrowth [54]. APP is highly expressed in the developing corpus callosum [55], where more than 200 million axons connect the two hemispheres [56]. *App*^−/−^ and *Aplp2*^−/−^ knockout mice are shown to have reduced in the density of myelinated axons in corpus callosum region [57]. Moreover, *App*^−/−^ mice fail to remyelinate after cuprizone-induced demyelination, which was not observed in *Aplp2*^−/−^ mice, suggesting that APPs regulate myelination differently [57]. In conditional triple APPs knockout mice, loss of all APPs after embryonic day 11.5 results in agenesis of corpus callosum [58]. A mechanism by which APP stimulates neurite outgrowth is via its interaction with reelin, which is known to regulate dendritogenesis [59–61]. The effect of reelin on neurite outgrowth is diminished in APP knockdown neurons [62]. Additionally, integrins, a class of transmembrane proteins that mediate the adhesion of the extracellular matrix and cytoskeleton, are proposed to be involved in APP–reelin-mediated neurite outgrowth as integrin has been shown to interact with these two proteins [62,63]. Indeed, blocking antibodies targeting α3β1 integrin reduces APP- or reelin–mediated dendritic outgrowth in neurons. As reelin has been demonstrated to enhance the interaction between APP and integrin, it has been suggested that the formation of an extracellular tripartite complex of APP, integrin and reelin triggers neurite extension [62].

Another extracellular molecule that may be associated with APP-mediated neurite outgrowth is F-spondin, a secreted morpho-regulatory protein. F-spondin interacts with the E2 domain in the extracellular regions of APP and APLPs via its reelin-like and spondin domains. F-spondin has been shown to promote neurite extension in several types of neurons including dorsal root ganglion (DRG) and commissural neurons [64,65]. Its role in stimulating neurite outgrowth is further supported by the incubation of DRG neurons with anti-F-spondin antibodies which suppresses F-spondin-mediated neurite outgrowth [66]. Conversely, F-spondin exerts an inhibitory effect on the outgrowth of motor axons [65]. These contrasting effects of F-spondin on neurite outgrowth may be due to the existence of different F-spondin interacting partners in different types of neurons. Of note, the protein-binding thrombospondin type 1 repeat 5 (TSR5) and TSR6 domains of F-spondin have been reported to enhance neurite outgrowth, whereas TSR1-4 domains suppress this process [67]. Therefore, it would be interesting to know the effect of F-spondin on neurite outgrowth after complexing with APP or APLPs.

Recently, APP has been reported to stimulate neuritogenesis via the extracellular signal-regulated kinase pathway by interacting with the EGFR ligands epidermal growth factor (EGF) and heparin-binding EGF-like growth factor (HB-EGF). The effects of APP and EGF on neurite outgrowth are abolished by treatment with an EGFR inhibitor [68].

In addition to the holoprotein, soluble APP fragments have also been found to participate in neurite outgrowth [69]. Additionally, sAPP has been found to interact with death receptor 6 (DR6) and trigger the DR6-dependent degeneration of sensory axons in commissural neurons [70]. Intriguingly, the *cis*-interaction of APP and DR6 has been shown to modulate the axonal pruning of retinal neurons which is a process for remodeling and extension of axons [71]. sAPPα is also required for depolarization-induced neurite outgrowth in neural stem cell-derived neurons through *N*-methyl-d-aspartic acid receptor (NMDAR)- mitogen-activated protein kinase (MAPK) pathway [56]. Of note, NMDAR–MAPK signaling is closely related to neuron survival and synaptic plasticity [72]. Abnormal NMDAR signaling has been implicated in the pathogenesis of AD [73]. As neurite degeneration is commonly observed in AD, it is worth investigating whether aberrant sAPPα/NMDAR functions contribute to AD pathogenesis.

### Axonal guidance

In addition to neurite outgrowth, axonal guidance is an important process in the developing nervous system. During the process, a growing axon is given signal(s) to reach a specific destination. Wang et al. demonstrated that APP, via the E1 domain, mediates axonal guidance through an interaction with Slit2, a member of the Slit family of guidance proteins. Importantly, axon repulsion induced by Slit2 is abolished in a three-dimensional olfactory explant culture derived from *App*^−/−^/*Aplp2*^−/−^ double knockout mice. The downstream effect of Slit2-APP on axonal guidance is thought to be triggered by p21-activated kinase 1 (PAK1) as the neuronal adaptor FE65 mediates the interaction of APP and PAK1. Such APP–FE65–PAK1 complex formation is potentiated by Slit2 [55]. However, it remains unclear how PAK1 is activated in the complex. It is noteworthy that exposure to recombinant Slit2 enhances the activation of Rac1, an upstream activator for PAK1. FE65 also binds to and activates engulfment and cell motility 1 (ELMO1)-dedicator of cytokinesis 1 (DOCK1) bipartite Rac1 guanine nucleotide exchange factor complex [74,75]. It is, therefore, worth investigating the roles of ELMO1–DOCK1 and/or Rac1 in this pathway.

Additionally, APP has been reported to serve as a co-receptor for guidance molecules such as Wnt family proteins which are vital regulators of various developmental processes [76]. For example, APP binds to the canonical Wnt receptor LDLR-related protein 6 (LRP6) to up-regulate the Wnt/β-catenin pathway [77]. Moreover, APP has been demonstrated to complex with DCC, a well-known receptor for the guidance molecule netrin-1, to regulate netrin-1-mediated axonal guidance in commissural neurons [78]. Recently, APP has also been proposed to function as a receptor of Wnt proteins as the cysteine-rich E1 domain of APP has been shown to interact with both Wnt3a and Wnt5a [76]. APP and Wnt3a function to stimulate the growth of axons. Conversely, the effect of Wnt5a on axon growth is promoted in primary neurons with an APP E1 domain deletion mutant which suggests an inhibitory role of Wnt5a-APP in the process [76]. It is possible that APP serves as a universal receptor for the Wnt family proteins to elicit different effects on axonal guidance.

Contactins are brain-enriched proteins belonging to neural immunoglobulin domain-containing cell adhesion molecules (CAMs). APP and APLP have been reported to interact with the contactins through its E1 domain [79–82]. In the developing retinotectal system, contactin-4 regulates the targeting of retinal ganglion cell axons to the nucleus of the optic tract [83]. Intriguingly, ectopic expression of contactin in APP-null mice failed to induce the targeting bias, suggesting that APP is indispensable for this process. Later research has identified that several contactins are able to interact with APPs in a *cis*-orientation, which may function as co-receptors during axon arborization in retinal ganglion cells [82]. It is proposed that APPs-contactins interaction may facilitate the intercellular interaction of another APP-contactin pair or other receptors on the cell surface [82].

Semaphorins (Sema) are diverse extracellular proteins that signals chemorepulsion and growth cone collapse. sAPPα has been shown to bind semaphorin 3A (Sema3A) [84]. Treatment with Sema3A induces growth cone collapse in DRG cells, which is inhibited by the presence of exogenous sAPPα [84]. Notably, the application of synthetic peptides containing the putative sAPPα-interacting sequence of Sema3A blocks the effect of sAPPα [84].

### Synaptogenesis

After a growing axon reaches its target, a synapse is required for the presynaptic neuron to transmit a nerve signal to the downstream cells. *App*^−/−^ knockout mice display an age-dependent decrease in the length and density of dendritic spines [85]. APP-deficient CA1 pyramidal neurons show reduced branching in dendrites [86]. The branching defect is exacerbated in combined *App*^−/−^*/Aplp2^−/−^* knockout mice [86]. Depletion of APLP1 decreases network inhibition and increases excitatory transmission [87]. In fact, *App*^−/−^*/Aplp2*^−/−^, *Aplp1*^−/−^*/Aplp2*^−/−^ double knockout and *App*^−/−^*/Aplp1*^−/−^*/Aplp2*^−/−^ mice die shortly after birth probably due to severe defects in neuromuscular synapses [24,88,89]. Cell–cell adhesion mediated by CAMs is a crucial step for synaptogenesis. It is believed that APP functions as a CAM in this process because the expression levels of APPs are increased during postembryonic synaptogenesis [90–92]. Moreover, the number of cell contacts are increased in the cells transfected with APPs [5]. Furthermore, excessive axonal terminal sprouting, a characteristic of defective synaptogenesis, is observed in *App*^−/−^/*Aplp2*^−/−^ double knockout mice [88].

In neurons, APP is transported along axons via kinesin-dependent anterograde transport and accumulates in synaptic sites [93,94]. In *Drosophila*, APP-like (APPL) was found to regulate the sorting of the adhesion molecule neuroglian, which has a role in synaptogenesis by organizing cytoskeletal molecules in synaptic terminals [95,96].

Dab1 is another APP interactor implicated in the formation of synapses. Dab1 also binds to Abl tyrosine kinase which is essential for synaptic remodeling [97]. It has been proposed that the effect of APP is triggered through the Dab1–Abl complex as the co-expression of kinase-dead Abl mutant reduces APP-induced axonal arborization [98].

On the other hand, APP is also transported to the postsynaptic terminal via dynein-mediated retrograde transport. In the dendrites, APP may also regulate synaptogenesis by sorting neurotransmitter receptors and CAMs to the synapses. A recent study showed that APP complexes with GBRs to deliver the receptors to the synaptic terminals as defective trafficking of GBRs is observed in *App*^−/−^ mice [99].

The interaction between APP and neurexins (NRXNs), a family of presynaptic adhesion proteins responsible for the formation and maintenance of synapses, is indispensable for synaptogenesis mediated by APP as the knockdown of NRXNs abolishes this process [100]. It has been proposed that dendritic APP binds and recruits NRXN-β on the axon surface and induces the clustering of NRXNs, which further triggers the recruitment of synaptic vesicles for presynaptic differentiation [100,101].

The mammalian neuromuscular junction (NMJ) is a specialized structure between a motor nerve and a muscle. APP is reported to localize to NMJ [102]. Mice depleted of APP and APLP2 exhibit abnormal NMJ formation and impaired synaptic transmission [88]. The effect of APPs on NMJ function is, at least in part, mediated by the FE65s, as the combined deletion of APLP2 with FE65/FE65L1 exacerbates NMJ deficits when compared with single deletion [103]. Additionally, loss of APP/APLP2 results in the absence of high-affinity choline transporter (CHT) in the presynaptic region. As APP-CTF interacts with CHT, it is proposed that APP functions to regulate the endocytic recycling of the transporter [104].

APP forms a presynaptic complex with X11 and calcium/calmodulin-dependent serine protein kinase (Cask) via AICD, and this complex stimulates presynaptic differentiation [105]. Of note, the X11–Cask complex has been proposed to be a common regulator of synaptic adhesion proteins during presynaptic differentiation through interaction with CAMs [105]. Moreover, in *Drosophila* NMJ, APPL complexes with the CAM fasciclin II to potentiate the effect of the CAM on new synapse formation. It has also been found that neither APPL with the intracellular domain deleted nor *Drosophila* X11 (dX11) with PTB domain deleted stimulates synaptic growth which suggests a role of the APPL–dX11 interaction in this process. It has been suggested fasciclin II, APPL and dX11 form a tripartite complex to transduce fasciclin II signals downstream to trigger synaptogenesis [106].

LRP4, a key regulator of NMJ formation, is another binding partner of APP [107]. In motor neurons, APP, agrin and LRP4 form a hetero-oligomeric complex and cooperatively amplify muscle-specific kinase activation, thereby, inducing the clustering of acetylcholine receptors on the postsynaptic membrane, a hallmark of NMJ formation [107].

## Conclusions

Despite of the central pathological roles of aberrant APP processing in neurodegenerative diseases, APPs also play crucial physiological roles in brain development. Many findings, as illustrated here, have demonstrated the significance of the interplay between APP and its interactors in the establishment and maintenance of the nervous system. Additionally, some APP interactors have been found to regulate APP processing which in turn mediates the release of bioactive APP fragments. For example, the interaction between FE65 and APP potentiates APP cleavage while X11 binds APP to suppress the process [11,13–16,108–111]. However, the precise effects of many APP interactors, including autosomal recessive hypercholesterolemia [112,113], on APP processing remain to be determined. Studies on such effects would provide new mechanistic insights into how these APP interactors participate in neurodevelopment by modulating the production of various APP cleavage fragments.

Notably, different APP processing pathways release structurally similar fragments. For instance, both sAPPα and sAPPβ consist of the protein-binding E1 and E2 domains, but they are reported to possess differential biological activities [114–116]. The only difference between the two secreted APPs is the presence of the 16-residues N-terminal Aβ sequence in sAPPα. Whether there is cleavage-specific binding partner(s) or target(s) is needed to be elucidated.

Knockout studies have revealed functional compensation, at least in part, between APPs [22–25]. As they share a similar domain structure, it is expected that the interactors of APP may also bind to APLPs to trigger related physiological responses. However, the unique functions of APPs in the nervous system, such as the differential synaptic roles of APP and APLPs in NMJ synapses, have also been reported [117]. The differential functions of APPs may be contributed by their recruitment of different interactors or differences in binding affinity toward the same interactor. Thus, detailed comparative studies of the interactions of APPs and their interactors are needed.

Differential expression of APP in various developmental stages and regions of the brain has been shown [19–21,92,118,119]. Likewise, the expression of APP interactors has also been found altered during development [120–122]. Of note, competition between interactors that bind to APP through the same region or domain is observed. For instance, FE65 and X11 compete for the binding of the AICD [123]. Although it remains unknown, the spatial-temporal expression and regulation of APP interactors at different neurodevelopmental phases may play a significant role in coordinating their interactions with APP. Moreover, our knowledge of how the differential binding of APP interactors is controlled remains limited. It is noteworthy that APP and some of its interactors are phosphoproteins. Many findings have revealed the crucial roles of protein phosphorylation in various neuronal processes [124]. Several reports have shown the importance of phosphorylation in modulating the bindings between APP and its interactors [14,36,125,126]. Further studies on the mechanisms that regulate APP interactions, including phosphorylation-dependent interactome analysis, would provide more mechanistic insights into how APP interplays with different interactors.

Although many APP interactors have been demonstrated to participate in different neuronal processes, such as FE65 in neurite outgrowth [74,75,127,128], their effects after binding to APPs remain elusive. Elucidation of the roles of these APP–interactor complexes would provide further insights into how these interactors participate in APP-mediated neurodevelopmental processes.

The generation of Aβ is a major focus in the study of the pathological roles of APP. However, evidence also suggests alternative pathological roles of APP. For instance, the D678N mutation on APP has been reported to cause AD-related dementia without a significant change in Aβ production [129] which suggests the impairment of other APP-associated processes. Notably, many neurodevelopmental processes that involve APP, including neurogenesis [130], neurite outgrowth [98,131] and synaptogenesis [132], are conserved in the adult nervous system. Defects in these neurodevelopmental processes have been observed in various neurodegenerative disorders such as AD and Parkinson's disease [133,134]. Targeting these processes that are mediated by APP–interactor complexes may be a novel avenue for the treatment of these disorders. Future investigations of the physiological roles of APP and its interactors would lead to a better understanding of their relationships with neurodegenerative diseases and would provide important information to facilitate the development of effective therapeutic interventions for these diseases.

## Perspectives

- There has been significant progress toward understanding the physiological roles of APP and its interactors in the development of the nervous system.
- The APP interactome has been implicated in modulating the diverse biological activities of APP. Identification of novel APP interactors is fundamental to increase our knowledge of the APP-regulated signaling.
- The linkage between APP physiological and pathological roles remains unclear. Elucidating how APP interactions contribute to normal neuronal functions offers insights into how APP may directly contribute to the pathogenesis of several neurological diseases.

## Open Access

Open access for this article was enabled by the participation of Chinese University of Hong Kong in an all-inclusive *Read & Publish* agreement with Portland Press and the Biochemical Society under a transformative agreement with JULAC.

## Abbreviations

AcD: acidic domain
AD: Alzheimer's disease
AICD: APP intracellular domain
APLP: APP-like protein
APP: amyloid precursor protein
APPL: APP-like
APPs: APP family proteins
Aβ: amyloid-β
CAM: cell adhesion molecule
Cask: calcium/calmodulin-dependent serine protein kinase
CHT: high-affinity choline transporter
CR: Cajal–Retzius
CTF: C-terminal fragment
CuBD: copper-binding domain
Dab1: Disabled-1
DISC1: disrupted-in-schizophrenia 1
DOCK1: dedicator of cytokinesis 1
DR6: death receptor 6
DRG: dorsal root ganglion
dX11: *Drosophila* X11
EGF: epidermal growth factor
EGFR: epidermal growth factor receptor
ELMO1: engulfment and cell motility 1
GBR: gamma-aminobutyric acid type B receptors
GEF: guanine nucleotide exchange factor
GFLD: growth-factor-like domain
HBD: heparin-binding domain
HB-EGF: heparin-binding EGF-like growth factor
JMR: juxtamembrane region
KPI: kunitz protease inhibitor
LDLRs: low-density lipoprotein receptors
LRP: low-density lipoprotein receptor-related protein
NMDAR: *N*-methyl-d-aspartic acid receptor
NMJ: neuromuscular junction
NPCs: neural precursor cells
NRXN: neurexin
Ox-2: Ox-2 antigen domain
PAK1: p21-activated kinase 1
PTB: phosphotyrosine-binding
RIP: regulated intramembrane proteolysis
sAPP: secreted APP
Sema: Semaphorins
T668: threonine 668
Tip60: Tat interactive protein 60kDa
TSR: thrombospondin type 1 repeat
YENPTY: tyrosine–glutamate–asparagine–proline–threonine–tyrosine
ZnBD: zinc-binding domain
